# Fighting in the legal grey area: an analysis of the German Federal Court of Justice decision in case preimplantation genetic diagnosis

**DOI:** 10.1007/s10202-011-0093-y

**Published:** 2011-06-28

**Authors:** Susanne Benöhr-Laqueur

**Affiliations:** 1 Zeppelinstr. 1, 27568 Bremerhaven, Germany; 2 School of Computing Science, Business Administration, Economics and Law, Department of Business Administration, Economics and Law, University of Oldenburg, Ammerländer Heerstr. 114-118, 26129 Oldenburg, Germany

## Abstract

According to the German Embryo Protection Act, PGD has been banned in Germany since 1990; one reason is the legislature’s avoiding to insert a revision clause regarding medical advance into the law. The ruling of the German Federal Court of Justice of July 2010 shows the problems resulting out of this approach and declares PGD to be permitted in certain cases. The article discusses the necessity for, as well as the problems of, an interdisciplinary dialogue in the field of reproductive medicine.

## Introduction

In 2005 and 2006, a Berlin-based gynaecologist genetically examined fertilised eggs from three couples for a predisposition to hereditary diseases. The doctor only implanted those that exhibited no genetic defects. The others were left to die. One of the three women became pregnant as a result, and gave birth to a healthy baby (Research in Germany 2010).

In January 2006, the doctor handed himself over to the authorities, because knowing full well that such a procedure could have legal implications. The central issue was whether *preimplantation genetic diagnosis* (PGD) was counter to the Embryo Protection Act of 1990. Now, the doctor turned to the courts for clarity (Hawley 2010). The Federal Court of Justice (BGH 2010) ruled in July 2010 that such procedures were not in violation to the Germany’s Embryo Protection Law. This opinion was shared by both the Berlin Regional Court (LG Berlin 2009), which dealt with the case in May 2009, as well as the Federal Court of Justice.

The verdict attracted great attention in Germany, as it sparked a new debate regarding a changing of the Embryo Protection Act. In this context, since late autumn 2010, three draft laws are being discussed across the parliamentary groups. The first of them provides a total ban on PGD (Schmidt et al. 2011). The second proposal limits PGD to cases of risk of miscarriage, stillbirth or severe genetic defects (Hintze et al. 2010). To prevent abuse consultation is to be mandatory, and an ethics committee has to agree. The woman has to express her acceptance in writing, and PGD is to be made only by licensed centres. The third draft law does not allow examination for possible genetic defects and limits PGD to cases of threat of miscarriage or stillbirth only (Röspel et al. 2010). In January 2011, the National Academy of Science has expressed its support for PGD (Nationale Akademie der Wissenschaften 2011). The decision of the German Ethic council was ambivalent. In March 2011, it decided in favour of PGD with the narrowest majority possible (Ethikrat 2011), and a votum of the parliament is expected in the summer of 2011 (Beck-Online 2011). Considering the volatile nature of the subject, it seems questionable whether this ambitious schedule can be kept.

## Reasons for the verdict

In its decision, the German Federal Court of Justice declared that an examination of pluripotent cells for serious genetical damages and their subsequent non-implantation, that is, their destruction, is not to be regarded as punishable (BGH 2010:ml. 37) and that PGD is not contrary to the Embryo Protection Act. An unlimited selection of embryos based on genetical characteristics for purposes, such as selection of sex, was still prohibited (BGH 2010:ml. 28).

In 1990, the legislators’ intention was to allow in vitro fertilisation only under the condition that its objective was to achieve a pregnancy. The government bill as well as statements outside the legislative procedure itself did only concern viable (‘totipotent’) cells. They were shaped by the fear of these cells being damaged resulting in effects on the life of the child being born after such a manipulation (BGH 2010:ml. 23). A PGD of totipotent cells, therefore, is clearly prohibited and punishable (BGH 2010:ml. 21). This does, however, not cover the intention to examine pluripotent cells for serious genetical defects. In the bill of 1989/1990, the legislator obviously did not envisage a PGD of pluripotent cells (BGH 2010:ml. 21).

This is the case, however, in the blastocyst biopsy carried out. From the blastocyst made up out of between 40 and 80 cells not anymore totipotent cells are taken, which at a later stage form the (child’s) endospern (placenta). Therefore, the embryo (-blast) itself is not affected. Such diagnostic methods were not available at the time the Embryo Protection Act was laid down. At that time, PGD was developed first in other countries. This explains why an expressed rejection or approval of such a PGD is to be found neither in the wording of the bill nor in the legislative records, though discussed in the procedure of legislation (BGH 2010:ml. 23, 24).

In other words: If a gynaecologist conducts a PGD with pluripotent cells in the blastocyst stage, this is permitted. But, if he would remove totipotent cells in the blastomere stage, 2–3 days after fertilisation, he would be liable to prosecution. This verdict is to be welcomed in the result, although it does give rise to various questions. First of all, it has to be clarified from what point on cells leave the stage of totipotence. Furthermore, the question arises why European reproductive centres still perform PGD in the 4- and 8-cell stage, though it can endanger the health of the embryo. Finally, it has to be clarified, what consequence a German gynaecologist’s transferring his patient to a European hospital, where this method is practised, can have.

## In the definition trap?!

### What is totipotence?

When and until what point is an embryonic cell to be classified as ‘totipotent’? The answer is more problematic than expected. By linguistic interpretation, the term derives from Latin *totus* (whole) and *potentia* (capability, force or power) (Taupitz 2008:§ 8, ml. 41). In biological terms, that means that such cells possess the potential to develop into an entire organism (Taupitz 2008:§ 8, ml. 41). As for human cells totipotence, this implies that they can develop into a complete human body (Taupitz 2008:§ 8, ml. 41). The question, however, is up to which point the cell possesses this quality. In this context, one can only rely on animal testing, which makes it most probable, that human cells lose their totipotence as soon as they have reached the 8-cell stage (Taupitz 2008:§ 8, ml. 43). In the 16-cell stage, this can be considered as a fact (Taupitz 2008:§ 8, ml. 43).

It can therefore be stated that most probably embryonic cells in the *blastomere stage* are totipotent, whereas in the *blastocyst stage* they have lost this quality. It is, however, a matter of fact that science is not in a position to determine the exact period of transition between totipotency and pluripotency of the human embryo (Taupitz 2008:§ 8, ml. 43).

### Practical application

Today’s medical possibilities, such as to biopsy in the 40–80-cell stage without damaging the embryo, where not foreseeable at the time of the passing of the Embryo Protection Act in 1990 (BGH 2010:ml. 24). Subject of discussion then was *blastomere biopsy* in 4- and 8-cell stage, which had been carried out successfully in England just before the entry into force of the law (Rose 2010). At that time, the possibility of the embryo suffering damage by PGD could not be excluded. Consequently, it was stated in the bill that at the time there was no reason to consider exceptions—as for preimplantation diagnosis—to the criminal prohibition (Bundesregierung 1989:11). These concerns were not shared by other European countries. It seems that since 1990, *blastomere biopsy* has become the preferred method in European hospitals (Fricke 2010). This is not without reason. The female cycle provides for the nidation of the embryo in an optimal manner. In artificial insemination, this natural process has to be re-enacted with difficulty and under time pressure (Nationale Akademie der Wissenschaften 2011:15). In *blastocyst biopsy,* the time for a nidation in many cases is missed; thus causing the necessity of cryoconserving the embryo until the next cycle (Nationale Akademie der Wissenschaften 2011:15). This is not necessary in a biopsy during the *blastomere stage* where the embryo can be transferred during the current female cycle. In addition, almost 20 years of experience indicate that the possibility of a damaging of the embryo can be excluded almost completely (Libaers 2010; Simpson 2010). For these reasons, not only the question of approval of PGD of ‘pluripotent’ cells is to be discussed but that of ‘totipotent’ cells as well, as today’s state of medical research has made the former objections obsolete. Therefore, the jurisprudence of the German Federal Court of Justice has created no legal clarity. The situation is that a German gynaecologist sending a patient to a hospital abroad for PGD, knowing that a blastomere biopsy in the 4–8-cell stage—i.e. with totipotent cells—will be preformed, is still potentially punishable (Däubler-Gmelin 2001).

In summary, it can be stated that terms such as ‘totipotency’and ‘pluripotency’,which are subjected to scientific progress in the long run, are not suitable to settle the issue adequately. Furthermore, a placement of the Embryo Protection Act in the (side-) criminal law is to be regarded as highly problematic. A solution of the dilemma in any case requires an interdisciplinary approach.

## Venture interdisciplinarity!

The Embryo Protection Law is a perfect example for demonstrating the tension between legal concepts and scientific progress. Unfortunately, jurisprudence is a subject particularly inaccessible for interdisciplinary contacts (Hilgendorf 2010:917). A reason for this is that the concepts and methods of jurisprudence have been developed so far and so very different from other subjects that interdisciplinary cooperation requires great effort (Hilgendorf 2010:917). Furthermore, lawyers tend to face imports from other disciplines sceptically, as modifications in the theoretical infrastructure of the legal system can easily have effects on the practical application and thus create the necessity of new dogmatic adjustments (Hilgendorf 2010:918). On the other hand, lawyers—for their own sake—must realise that regulations in the area of modern reproductive medicine can only be achieved interdisciplinarily as the analysis of a standard normally allows more than one interpretation. In order to decide which of them is the one to choose, empirical knowledge has to be considered, and this must be provided by the different disciplines (Hilgendorf 2010:920; Lendi 2009:178). When the legislator in 1990 equated the embryo with the ‘totipotent’cells, in spite of him being aware of the rapid progress of IVF technique worldwide, he deprived himself of the possibility of an interdisciplinary amendment.

To complicate the matter, the Embryo Protection Law was designed as criminal law. This means that crucial importance is given to the principle of certainty according to Art. 103 II GG: An act may be punished only if it was defined by a law as a criminal offence before the act was committed. If the legislator—as in this case—uses terms like ‘totipotence’, which are undergoing a constant change due to scientific progress, he unnecessarily steps into a ‘definition trap’.

All this leads to the conclusion that instead of amending the outdated Embryo Protection Act, a complete revision of this legal matter is to be considered. In this context, priority ought to be given to the question whether this legal material is to be sanctioned by criminal law at all (Taupitz § 8:ml. 43). The highly effective British Human Fertilisation and Embryology Act as well as the HFEA (Human Fertilisation and Embryology Authority) are examples demonstrating how a piece of legislation can be opened for non-juridical standards by an appeal to ‘to the state of the art’and by creating a regulatory authority (Benöhr-Laqueur 2007).
